# The modal status of the laws of nature. Tahko’s hybrid view and the kinematical/dynamical distinction

**DOI:** 10.1007/s13194-020-00335-4

**Published:** 2021-01-11

**Authors:** Salim Hirèche, Niels Linnemann, Robert Michels, Lisa Vogt

**Affiliations:** 1 Département de philosophie, Université de Genève, 2 rue de Candolle, CH-1211 Genève 4, Switzerland; 2 Institut für Philosophie, Universität Bremen FB 9, Postfach 330 440, Enrique-Schmidt-Str. 7, D-28334 Bremen, Germany; 3 Western Interdisciplinary Research Building, Rotman Institute of Philosophy, Western University, 1151 Richmond Street North, London, Ontario CA-N6A5B7 Canada; 4 Facoltà di scienze della comunicazione, Università della Svizzera italiana, Via G. Buffi 13, CH-6900 Lugano, Switzerland; 5 LOGOS, Facultat de Filosofia, Universitat de Barcelona, Carrer de Montalegre 6-8, ES-08001 Barcelona, Spain; 6 Institut für Philosophie, Freie Universität Berlin, Habelschwerdter Allee 30, DE-14195 Berlin, Germany

**Keywords:** Hybrid law account, Essence, Laws of nature, Modality, Metaphysical necessity, Naturalised metaphysics, Kinematical/dynamical distinction, Physical laws

## Abstract

In a recent paper, Tuomas Tahko has argued for a hybrid view of the laws of nature, according to which some physical laws are metaphysically necessary, while others are metaphysically contingent. In this paper, we show that his criterion for distinguishing between these two kinds of laws — which crucially relies on the essences of natural kinds — is on its own unsatisfactory. We then propose an alternative way of drawing the metaphysically necessary/contingent distinction for laws of physics based on the central kinematical/dynamical distinction used in physical theorising, and argue that the criterion can be used to amend Tahko’s own account, but also that it can be combined with different metaphysical views about the source of necessity.

## Introduction

The laws of nature are nomologically necessary, but are they also metaphysically necessary? The three theories which dominate the recent discussion about the metaphysics of laws of nature give different answers to this question: according to *Dispositional Essentialism* they are (see e.g. Bird, 2007; Ellis, 2001), according to the *Nomic Necessitation View* (see e.g. Armstrong, 1983; Dretske, 1977; Tooley, 1977) and *Humeanism* (see e.g. Lewis, 1973) they are not.

What these theories have in common is that they are *absolutist*: they tell us that all laws of nature have the same metaphysical modal status; they are either all metaphysically necessary, or all metaphysically contingent. Tuomas Tahko (2015) questions the current absolutist dogma and defends a view, according to which ‘[t]here is a middle ground between the two extreme views about the modal status of laws: a hybrid view, according to which some laws are contingent and some laws are necessary.’ (Tahko, 2015, p. 513) Tahko supports his hybrid view by proposing a criterion for drawing the distinction between metaphysically contingent and necessary laws which is based on a metaphysical view of natural kinds inspired by Lowe (2005). This criterion is then applied to physical laws, and to Coulomb’s law and the Pauli Exclusion Principle (PEP) in particular. His core idea is that the metaphysically necessary laws are those which feature fundamental natural kinds, while the contingent laws are those which do not. His paper is written in the spirit of contemporary naturalistic metaphysics, involving principled metaphysical reasoning, but also aiming at paying close attention to the relevant physics.

A crucial ingredient of Tahko’s argument for the hybrid view are discussions of particular physical laws which illustrate how the contrast between metaphysically necessary and contingent laws can be drawn using his natural kind-based criterion. In this paper, we will argue that this criterion on its own is insufficient, since it neither gives us a clear, nor stable, let alone science-informed indicator of the metaphysical modal status of laws. Based on this critique of Tahko’s criterion, we will then propose an alternative way of drawing the metaphysically necessary/contingent-distinction for laws of physics, which is based on an important distinction in physical theorising — the distinction between kinematical and dynamical structure. Our main aim is hence to vindicate Tahko’s hybrid, or anti-absolutist position by proposing a more robust way to draw the line between metaphysically necessary and contingent laws of physics, following the same naturalistic approach as Tahko.

The structure of the paper is as follows. In the next section, we focus on Tahko’s kind-based criterion, critically examine his two main case studies of physical laws and raise three problems for his criterion. In the third section, we introduce an alternative criterion which is based on the kinematical/dynamical distinction, and show how it can be applied to vindicate Tahko’s claim that Coulomb’s law is metaphysically contingent, while the PEP is metaphysically necessary. In the fourth section, we discuss whether our criterion can be seen as a refinement of Tahko’s, or whether it should be seen as a genuine alternative to it. The fifth section contains concluding remarks.

## Tahko’s kind-based criterion

Tahko (2015) argues for two main claims. The first concerns the modal status of the laws of nature. According to him, although the three dominant views are absolutist — attributing the same modal status to all laws — a closer look at particular examples from physical theories rather suggests a hybrid view: some laws are metaphysically necessary, while others are only nomologically necessary. Tahko’s second main claim concerns how this modal divide should be *explained*. His proposed account relies on a distinction between laws that involve fundamental natural kinds and laws that do not. More precisely, he claims that the metaphysically necessary laws are those that ‘feature’^1^ fundamental natural kinds, while the merely nomologically necessary laws are those that do not feature any fundamental kind (but perhaps natural properties instead; see Tahko (2015), p. 519). Our worries with Tahko’s overall position do not concern his first claim, but his second claim: even if he is right that current physical theories suggest a modal divide among laws of nature, his proposed account of this divide raises a number of issues.

First, his kind-based criterion does not offer a clear and robust way to divide laws into two distinct categories (the metaphysically necessary and the merely nomologically necessary laws). According to Tahko, the Pauli Exclusion Principle (PEP) — which states that no two fermions in a closed system can occupy the same quantum state at the same time — provides a typical example of a metaphysically necessary law, because it clearly features a fundamental kind — fermion.

By contrast, Tahko argues that Coulomb’s law — which quantifies the magnitude of the electrostatic force between two stationary electrically charged particles — provides a typical example of a law that is merely nomologically necessary, because it does not feature any fundamental kind. Yet, this may be disputed: one may also understand Coulomb’s law as featuring a fundamental kind — say, that of a material body. Tahko considers this option but (following Bird 2012) questions its plausibility on the grounds that the proposed candidate kind is too general — a ‘kind’ whose members include *all* material bodies, he argues, would be ‘a peculiar kind indeed’ (p. 516).

However, it is certainly not obvious that generality should be a crucial criterion in deciding whether something is a fundamental kind in the first place. And in any case, the candidate kind just considered is not the only one available: Coulomb’s law may be read as featuring the kind *charged body*/*being charged*. This alternative candidate is arguably more plausible as such: Coulomb’s law applies to all charged bodies rather than to all material bodies in general. More importantly, this candidate does not face the alleged generality problem — at least not to the same extent —, and it is not clear what reason could be invoked to convincingly exclude it.^2^

Overall, the example of Coulomb’s law illustrates a more general point: in many cases, the question whether a given law of nature features a (fundamental) kind will presumably be subject to disagreement, and it is hard to see how the question could be settled in a principled way.

A second, independent problem with Tahko’s proposed account over and above the just discussed division problem concerns its ability to indeed *explain* the modal divide between metaphysically and merely nomologically necessary laws. For suppose that this first problem does not arise: we can clearly distinguish between laws that feature a fundamental kind, and those that do not; and the laws of the first sort are all metaphysically necessary, while those of the second sort are all merely nomologically necessary. Now, how is a distinction between ontological categories (fundamental kind versus property or non-fundamental kind) supposed to explain a distinction between two modal forces?^3^ The former distinction seems irrelevant to the latter; if indeed there were a perfect correspondence between the two, it would look like a surprising coincidence. Consider how Tahko himself describes his preferred illustration of the claim that metaphysically necessary laws feature a fundamental kind:

[I]t is part of the nature of fermions that they behave in a manner that is constrained by the PEP. … [C]learly, the fact that the behaviour of fermions is constrained by the PEP is at least partly due to their half-integer spin … [I]t is also plausible that half-integer spin is essential for fermions… (Tahko 2015, p. 524)

The fact that fermions constitute a fundamental natural kind rather than e.g. a natural property or non-fundamental kind plays no role here – indeed, it is completely absent from the picture. The PEP owes its modal status entirely to its being *essential* to fermions. Thus, even assuming that Coulomb’s law features no fundamental kind – say, because being an electrically charged body is a natural property instead –, it may be argued, by similar reasoning, that the law is still metaphysically necessary: it is part of the essence of the property of being an electrically charged particle that all of its instances behave in a way that is constrained by Coulomb’s law. One may of course deny that it is essential to charged particles that they obey Coulomb’s law, and deny *on that basis* that the law is metaphysically necessary. The point is that the distinction between metaphysically necessary and contingent laws relies on the distinction between laws that are essential to the entities that they govern, and those that are not – whether those entities are fundamental kinds, or non-fundamental kinds or natural properties instead. Everyone who works with the notion of essence in contemporary philosophy can agree that essentiality implies metaphysical necessity, no matter which of the two dominant metaphysical views about essence they accept, the modal view, according to which to be essential just is to be metaphysically necessary, or the primitivist view about essence (see Fine 1994a); this is not the issue at stake here.^4^ The real question is rather the following: what accounts, in turn, for the distinction between essential and non-essential laws — why think that the PEP is essential to what it governs, while Coulomb’s law is not? Tahko’s kind-based criterion can hardly be the answer, absent some further assumption about kinds being (unlike e.g. natural properties) related to essences in a privileged way.

Our third worry about Tahko’s account is methodological. Tahko advocates the view that metaphysics should take our best scientific theories into account. Indeed, as pointed out earlier, his defence of a hybrid view about the modal status of laws relies on concrete examples drawn from physical theories. One would expect him to take the same methodological stance when looking for an account of this modal divide. Yet, the distinction between laws that feature a fundamental kind and laws that feature a natural property can hardly be considered as motivated by scientific practice or scientific theories — such ontological distinctions are not central, or even common, in physics.

In sum, although Tahko may be right that physics gives us reasons to adopt a hybrid view of the modal status of laws, his proposed account of this modal divide, based on his proposed kind-based criterion, raises a number of problems.

## A criterion based on the kinematical/dynamical distinction

In this section, we propose and defend an alternative account to Tahko’s purely metaphysical criterion which avoids those problems. It is based on what is known as the kinematical/dynamical distinction in physical theorising and it (1) allows for a clearer division of laws into two categories, (2) is relevant to explain the modal difference between them, and (3) is directly drawn from physics.

All modern physical theories explicitly dealing with physical processes — i.e. classical theories, (general) relativistic theories and quantum mechanics^5^ — draw on a distinction between kinematics and dynamics.^6^

Following Curiel (2016), kinematics is about (i) classifying physical quantities into quantities which are constant over time and quantities which change over time, (ii) characterising the notion of a physical state adhered to in the theory through recourse to these two sorts of quantities, and (iii) setting up further constraints on the space of allowed states. Concerning the latter, Curiel distinguishes between local and global kinematical constraints. Local kinematical constraints are constraints that concern single but generic states. Examples for such constraints include the Heisenberg uncertainty principle ${\Delta } x {\Delta } p \geq \frac {1}{2} \hbar $ for position *x* and momentum *p*, and the constraint that the shear-stress tensor in the hydrodynamic theory of Navier-Stokes is symmetric (to name a more technical example) (cf. Curiel 2016, p. 3). Global kinematical constraints are constraints associated with more than a single state. An example for such a constraint is Kepler’s Harmonic law, which states that the semi-major axis of a planet’s orbit is directly proportional to the square of its orbital period.

A general way to think of the kinematical/dynamical distinction is in terms of the kinematical structure as setting up the overall stage with respect to which specific dynamical behaviour can then be defined. In particular, kinematical structure refers to specific relations^7^ between physical quantities which are invariant across all possible models of a physical theory. Dynamical structure, by contrast, varies across all possible models of the theory/theory framework (cf. Curiel 2016).^8^

At least on Curiel’s formulation, one can thus naturally understand the distinction between kinematical and dynamical as involving a modal aspect: kinematical constraints give rise to a stronger sort of necessity — or equivalently, to a broader space of possibilities. A physical system *must* evolve in the way prescribed by dynamical laws; but the sense in which that system *must* conform to the relevant kinematical constraints is a stronger one. This modal aspect of the kinematical/dynamical distinction may then more precisely be interpreted in at least two different ways, one ontological, and one semantic in nature: kinematical structure consists of specific relations between physical quantities which are invariant across all ontologically/semantically possible models. In contrast, dynamical structure consists of specific relations between physical quantities which vary across ontologically/semantically possible models.

To be more specific, on the ontological interpretation, the sort of necessity attached to kinematical structure is what is commonly called metaphysical necessity; and accordingly, dynamical structure is metaphysically contingent. By contrast, on the semantic interpretation, kinematical constraints are semantically or conceptually necessary — they are ‘analytic’, i.e., required by the very definitions or meanings of the terms of the theory. And accordingly, dynamical structure is semantically contingent.

The generic core claim introduced earlier—that the kinematical/dynamical distinction aligns with a distinction between two modal statuses of different strength—remains exactly the same for both interpretations. What they differ over is rather how this generic claim is to be substantiated in terms of a particular *kind* (ontological or semantic) of modal status.

Curiel himself promotes a semantic view of the kinematical/dynamical distinction (see Curiel 2016) which distinguishes kinematical constraints from dynamical constraints in that the former are minimally required for the physical terms of the theory to have a meaning:

One may say that a theory has *propriety of representation* for a system when the system satisfies its kinematical constraints, for their satisfaction is semantically prior to the satisfaction of the equations of motion[…]. It therefore seems promising to attempt to base a semantics for physical theory on this idea:

We know the meaning of a theory when we know the conditions under which the kinematical constraints hold, i.e., when the the theory has propriety in representation. (Curiel 2016, p. 10-11)

Given Curiel’s semantic reading, this entails that the kinematical/dynamical distinction of a theory distinguishes its semantically necessary from its semantically contingent claims, i.e. those of its claims which *must* be kept fixed in order to conserve the meaning of the physical terms of the theory and those which *can* be changed without changing these meanings.

Yet, the way in which Curiel presents the distinction also leaves room for the mentioned alternative, *ontological* understanding of the stronger modality attached to kinematical constraints. And, as we will argue, given this alternative reading, the distinction can naturally serve as a basis for the sort of hybrid view of laws discussed earlier, according to which some laws are metaphysically necessary and others are merely nomologically necessary.

After all, according to Curiel, one main role that kinematical (but not dynamical) constraints play in physical theory is that ‘they characterise the physical *nature* of systems the theory treats, i.e., [they are] *constitutive* of the kind of system the theory treats’ (Curiel 2016, p. 9; emphasis added). Given that Curiel advocates a semantic interpretation of the kinematical/dynamical distinction, these claims about the nature of physical systems and about something’s being constitutive to them are arguably meant as claims about the meaning of physical terms. However, his formulation is at the same time highly suggestive of an essentialist reading:^9^ both ‘nature’ and ‘constitutive’ are staples in the contemporary essentialist’s vocabulary (cf. Fine 1994a). More precisely, assuming an ontological re-interpretation of Curiel’s take on the distinction, we may say that the kinematical constraints capture those aspects of a system which make it the sort of physical system it is according to the theory, paralleling almost exactly the Aristotelian characterisation of essence as the ‘what it is to be’, or the ‘real definition’ of an entity.^10^ Kinematical constraints in a physical theory hence play exactly the same role which essential characteristics of entities play in metaphysical theorising. Given the standard assumption that essentiality entails metaphysical necessity (cf. Fine 1994a), this systematic parallel strongly suggests that kinematical constraints are metaphysically necessary.^11^

This essentialist (re-)interpretation is not merely suggested by Curiel’s discussion of the distinction; it also perfectly fits the essentialist approach to modality which is accepted both by Tahko and by proponents of the dispositional essentialist theories of the laws of nature which provide the theoretical background for the development of his hybrid view (cf. e.g. Bird, 2007; Ellis, 2001). According to this approach, the question of whether a law is necessary or contingent is a question about its *metaphysical* necessity or contingency. It concerns what our universe and the things it contains could and must objectively be like, independently of how we describe it (cf. Kripke 1980). There is a mismatch between this objective, realist notion of modality and the meaning-based notion of modality associated with the kinematical/dynamical distinction according to the semantical interpretation. The latter notion is tied to a space of semantical or conceptual possibilities which exceeds that of the metaphysical possibilities (think of the well worn example of water’s being an element, which is metaphysically impossible, but conceptually possible).

Our main point in this section is that the kinematical/dynamical distinction can fruitfully be applied in the context of the debate about the metaphysical modal status of the laws of nature, providing us with an improved criterion for the metaphysical necessity of the laws of physics. In order to make this point, we will hence work with the ontological, essentialist interpretation of the distinction in what follows.^12^

We will now argue that Tahko’s paradigm examples for metaphysical necessary laws/contingent laws are readily accounted for on the basis of the dynamical/kinematical distinction. Since the distinction is well-established in physics, this clearly evades issue (3) of Tahko’s account.

But more than that: The division is clear and epistemologically accessible (evading issue (1)), and naturally conceived as relevant for a modal distinction (evading issue (2)), or so we will argue now.

Concerning the division problem (issue (1)): Coulomb’s law clearly counts as a dynamical law. The PEP, in contrast, is a kinematical law: remember that the PEP states that “no two fermions in a closed system can occupy the same quantum state at the same time” (Tahko 2015, p. 514). Now, fermions are all half-integer spin particles (as opposed to bosons which are all integer spin particles); the classification of particles into spin particles is a purely kinematical result (this follows from the Peter-Weyl theorem when assuming that the (kinematical) state-space is rotationally invariant^13^). The PEP is then the mere posit that certain spin-state combinations should be excluded. Thus, in the case at hand, the proposed criterion yields a clear-cut verdict concerning the modal statuses of the two laws, which illustrates that the criterion does not suffer from the epistemic intractability that we found with regard to Tahko’s proposal.

Concerning the modality problem (issue (2)): As suggested earlier, on the assumption that kinematical laws describe the physical aspects of the essences of entities, and given that essence entails metaphysical necessity — an assumption which, as noted before, is widely shared among contemporary metaphysicians —, it follows that the kinematical laws have the status of metaphysical necessities. Dynamical laws, by contrast, are concerned with the (metaphysically contingent) development of physical entities within the broader space of possibilities determined by the kinematical laws. This, together with the assumption that nomological necessity is a strictly weaker kind of necessity than metaphysical necessity and that nomological necessity is the kind of necessity associated with the laws of nature, suggests that the dynamical laws are nomologically, but not metaphysically necessary.

A worry that might seem to arise for the emerging picture of a natural alignment of the distinctions between kinematical/dynamical and metaphysically necessary/merely nomologically necessary laws is that the former is explicitly theory-relative, whereas the latter is traditionally considered to be theory-independent. Does this mean that our criterion is revisionary regarding this aspect of metaphysical modality? In one sense, it is: the criterion implies that if there were to be a change in how the line between what is part of the fundamental kinematical and dynamical structure is drawn in a future physical theory, this change would result in a shift in what we have to regard as metaphysically necessary and nomologically necessary. Even though there is currently no good reason to think so, in case it e.g. did turn out that the PEP described dynamical aspects of physical reality after all, we would accordingly have to retract our earlier claim about its modal status.

In another (arguably more important) sense, our criterion does not revise the conventional idea of metaphysical modality: when we make the idealising assumption that the distinction between kinematical and dynamical laws as it is drawn in *current* physical theories coincides with the distinction as drawn in a *final* physical theory which correctly represents the physical aspects of reality, we couple metaphysical modality to a specific theory after all. Nevertheless, we do take it to be already a fundamental posit of contemporary metaphysics, starting in particular with Kripke’s ground-breaking discussion of metaphysical necessity in Kripke (1980), that the laws of such a final theory reveal general physical features of our world which are essential to entities in it, features which are objectively necessary in the sense that they rule out any seemingly possible scenario which involves violations of these laws. Drawing once again on the connection between essence and metaphysical modality, we can note that these features are metaphysically necessary aspects of reality.^14^ It nearly goes without saying — given the idealizing assumption, and in light of the preceding discussion — that the particular claims about the metaphysical necessity or contingency of certain laws of current physics which we accept in this paper should be taken to be hypothetical, in the sense that they are informed by what we currently take to be our best physical theories.

What we are prepared to commit ourselves to, however, based on extrapolation from current and past successful physical theories, is the following: any future best physical theory will still involve the kinematical/dynamical distinction, and this distinction will still play the same theoretical role, thus offering us the best physical basis for drawing the distinction between metaphysically necessary and contingent laws.

It is worth stressing that all of this does not put us is in a worse spot than other naturalistic positions in metaphysics. To see this, think e.g. of Tahko’s original proposal and the imagined possibility that future physics might reveal that what we now call fermions are really a gerrymandered assortment of fundamentally different particles, disqualifying them from forming a fundamental physical kind.

Our overall argument for taking the kinematical/dynamical distinction from physics to provide us with a criterion for the metaphysical necessity of laws can be summed up as follows then:

1. The kinematical laws of a physical theory give us the properties which are *constitutive* of the physical systems involved relative to that theory. (Definition/characterization of kinematics).
2. The kinematical laws of a physical theory give us the properties which are *essential* to the physical systems involved relative to that theory. (Ontological/essentialist interpretation of the constitutive aspect of kinematics).
3. The kinematical laws of a *final* physical theory give us the properties which are essential to the physical systems involved. (Idealization assumption.)
4. Essentiality entails metaphysical necessity. (Widely accepted assumption in metaphysics following Kripke and Fine).
5. The dynamical laws of a final physical theory capture non-essential, but still in some other sense necessary aspects of the physical systems characterized by it. (Based on the two facts that (1) there are distinct ways in which these dynamical laws may be set up given the kinematical laws of a theory, and that (2) they have to allow for a range of different, and thereby contingent developments of the system over time. To be even more explicit: we assume here that contingency implies non-essentiality, which follows from the previous premise plus the standard assumption that necessity and possibility are duals).
6. That other sense of necessity is that of nomological necessity. (By the commonly accepted definition of nomological necessity as the modal force shared by all laws of nature).
7. The kinematical laws of a final physical theory are metaphysically necessary, whereas its dynamical laws are metaphysically contingent albeit still nomologically necessary.

## Necessity criteria and sources of necessity

How does our criterion relate to the kind-based criterion on which Tahko’s argument for the hybrid view is based? To respond to this question, it is helpful to distinguish three questions which one should be able to answer about the metaphysical necessity of laws of nature if one accepts a hybrid view.

1. *Which* laws are metaphysically necessary?
2. *How* can we determine which these laws are?
3. *Why* do these laws have this modal status?

Before we further discuss these questions, we should mention an important related question, namely that of what makes a law a law in the first place. Our aim in this paper is not to provide a full answer to this question. In particular we want to stress that our criterion for the metaphysical necessity of the laws, contrary to what one might think, does not do double duty as a criterion for lawhood: we claim that laws of nature are metaphysically necessary if they express kinematical constraints; we do not claim that any kinematical constraint expresses a law of nature.^15^ A fully developed theory of the laws of nature which includes our criterion will of course have to provide an answer to this question, but developing such a theory is not the task we have set ourselves in this paper.^16^

We evidently agree with Tahko’s answer to the *which*-question regarding all relevant examples he considers: we agree with him that PEP is metaphysically necessary, and that Coulomb’s law is metaphysically contingent. It is also evident that we disagree with him about the *how*-question, which we take to be an epistemic question about how we can find out that a particular law is metaphysically necessary: for the reasons given in Sections 2 and 3, we believe that our criterion gives us a better way to determine whether a law is, or is not metaphysically necessary.

In this section, we will focus on the third question. We have already made clear in section 2 that Tahko’s reliance on kinds does by itself not help much in answering the *why*-question: on his account, the real work in explaining the necessity of the metaphysically necessary laws is done by essences, not by kinds themselves.^17^

This is not to say that we think one cannot at all rely on kinds in defending a hybrid view. The point rather is that reliance on kinds as the carriers of essence is not as crucial to Tahko’s account as he makes it out to be. The crucial ingredient needed is an *essence-bearer* of some sort, be it a kind, a (natural) property (Shoemaker, 1980; Shoemaker, 1998; Kistler, 2002, 2005, 2020; Bird 2007), or some other entity. We will rely on this ontologically neutral term in the following discussion.

Given the tight connection between kinds and essence, Tahko’s answer to the *how*-question entails or at least very strongly suggests a particular answer to the *why*-question. Our proposal is different in this regard. Its starting point is a particular answer to the *how*-question, which in principle is compatible with different answers to the *why*-question, i.e., the question of the source of the metaphysical necessity of some laws.

This is not to say that we think that the core idea of Tahko’s answer to the *why*-question is unacceptable or incoherent, as long as the focus is broadened from kind essence to essence in general: an essence-bearer-based answer along the line of Tahko’s is a live option worth considering, which should be evident from the particular essentialist development of the hybrid view proposed in the previous section. Indeed, if one understands Tahko this way, one can treat our criterion as an amendment of Tahko’s original proposal which keeps his answers to the *which*- and *why*-questions, but replaces his answer to the *how*-question. A view of this form would obviously still support his hybrid position: it would say that only kinematical laws can express essential truths about physical entities and that dynamical laws cannot. Accordingly, Coulomb’s law would be classified as metaphysically contingent, since it concerns dynamical aspects of physical reality, whereas the PEP would turn out to be metaphysically necessary since it concerns exclusively kinematical aspects of physical reality.

It is straightforward to see that this amended version of Tahko’s position does not fall prey to the three problems that we raised for his original account in section 2: First, it gives us a robust criterion for which laws are metaphysically necessary and which are not. The lack of epistemic accessibility of Tahko’s (purely) kind-based criterion is eliminated, since the kinematical/dynamical distinction gives us a solid basis for determining whether or not a law is due to the essence of some entity, and thus metaphysically necessary.

Second, the proposed view makes it clearer that what accounts for the metaphysical necessity of certain laws, namely kinematical laws, is not their involving natural kinds as such, but really their expressing *essential* constraints on the entities that they govern — essentiality being in turn widely accepted as implying metaphysical necessity.

Third, the position is obviously more in line with the general programme of a naturalistic metaphysics, since it crucially relies on a relevant distinction which is very well entrenched in physical theories.

But as we have already mentioned, using the proposed criterion to amend Tahko’s account is not the only option, given that it only explicitly invokes the kinematical/dynamical distinction, but not the concept of essence: the criterion could in principle be combined with different answers to the ‘why’-question, i.e, with different views about the source of the metaphysical modality of the kinematical laws – such as counterfactual views (Williamson 2007), primitivist views (Forbes 1989), conventionalist views (Sidelle, 1989; Sider, 2011), or possible worlds views (Lewis 1986). Recall, however, that the motivation which we provided for thinking that the kinematical/dynamical distinction aligns with the metaphysically necessary/nomologically necessary distinction does rely on essence. Hence, although there is the option of endorsing our criterion while eschewing essence, the resulting theory would have to be supplemented by a different argument connecting the kinematical/dynamical distinction to the chosen source of necessity. Indeed, one may try to relate the kinematical/dynamical distinction to the distinction between metaphysical and nomological necessity in a more direct way, along the following lines: as we have suggested earlier, kinematical laws express constraints that are in some sense stronger, or stricter, than dynamical laws; and this may already be enough to suggest a modal divide: kinematical laws, accordingly, determine a stronger sort of necessity, or a broader space of possibilities.

In a similar vein, although it is our view that the divide in modal strength given a hybrid account is best conceived as one in terms of metaphysically necessary vs. merely nomologically necessary laws, we would like to note that there is in principle room for a hybrid view based on the kinematical/dynamical distinction that would construe the different modal forces as two grades *within* nomological necessity. This divide might then in turn be understood, for instance, as two degrees of stability under counterfactual variation (see Lange 2009). Such a view would clearly differ even further from the one advocated by Tahko, while being less revisionary with regard to the ‘orthodox view’.

So, all in all, while we do favour the variant of the view which ties the distinction between kinematical and dynamical laws to the distinction between metaphysical and nomological necessity and relies on the idea that essence is the source of the metaphysical necessity of the kinematical laws, we want to stress that there is a variety of other ways to flesh out variants of a hybrid view of the modal status of the laws based on our proposed criterion.

## Conclusion

Tahko’s view makes the metaphysical necessity of physical laws dependent on whether or not they feature particular kinds. It is, however, not clear how these putatively relevant kinds are picked out, and, as we have argued, kinds can in any case only play a mediating role as entities whose essences can account for the metaphysical necessity of a law. Furthermore, the relation between the metaphysical idea to posit an ontology of natural kinds and physical practice is at best unclear, which means that physics provides us with no direct justification for Tahko’s choice. In order to evade all three issues, we propose to adhere to the kinematical/dynamical distinction well-familiar from physical theorising: thereby, we are able to sort laws into those which are metaphysically necessary and those which are metaphysically contingent, giving us a hybrid account of the modal status of the laws which is in line with physical practice. At the same time, this account naturally suggests an essentialist view on which a physical law can only count as metaphysically necessary if it is due to what one might call the kinematical essence of some entity. This amendend version of Tahko’s account thereby evades all of the three concerns raised. We have, however, also pointed out that our criterion for drawing the line between metaphysically necessary and contingent laws is ultimately compatible not only with different views about the source of metaphysical modality, but more generally with different ways to flesh out a hybrid view about the modal status of the laws of physics.

## Footnotes

1 It is not entirely clear what ‘feature’ means in this context. See also footnote 17.

2 As a reviewer pointed out to us, however, what we take to be bodies without charge could instead be conceived as a specific sort of charged bodies — ones with charge zero. In this case, *charged body* would seem to be as general a candidate kind as *material body*. Two remarks are in order here: first, taking *charged body*, as a candidate kind for Coulomb’s law, to also include bodies of charge zero is indeed a possible option. Yet, it may be disputed. Although we do not deny that, for certain properties, having a zero-value for them might still intuitively count as having them in a way, charge strikes us as not belonging to this category. The same could be said about mass: a particle whose value for mass is zero is just a particle without mass. To put it otherwise, such physical properties strike us as being analogous, in that respect, to less fundamental properties like that of having money, for instance: if someone has zero euro, it would somehow be weird to insist that they still have money — a zero-amount of it. Rather, we would simply say that they don’t have money. Second, even assuming that Coulomb’s law plausibly applies to the kind *charged body*, that this kind in fact also covers bodies of charge zero, and that this would ultimately make this candidate kind as general as the candidate rejected by Tahko for its full generality, namely *material body*, this would not be a serious threat to our argument. For one thing, as we have noted, it is not that clear that generality should be an important criterion for kindhood in the first place. Moreover, and importantly, our primary goal here is not to argue that *being charged*, beyond any reasonable doubt, is the right candidate kind for Coulomb’s law, or that it is not fully general. Rather, we precisely use this example to illustrate how the question of whether a law features a kind or not may often be subject to reasonable disagreement.

3 An analogous worry arises when basing the distinction between necessary and contingent laws on that between natural and non-natural properties, as for instance done by Mellor et al. (1991).

4 See Kripke (1980) which established the notion of metaphysical necessity in contemporary anglophone mainstream philosophy and more recently the highly influential essentialist theory of modality, first proposed in Fine (1994a).

5 As opposed to say thermodynamics, which is first of all a theory of equilibrium states, and only indirectly makes statements about transitions between states.

6 Note that we are *not* using the notions of kinematics and dynamics in the sense of the specific sub*disciplines* of (classical) mechanics but in reference to kinematical and dynamical *structure* of a physical theory more generally (as also usually referred to outside the context of classical mechanics). Thanks to an anonymous reviewer for pressing us to clarify this point.

7 In contrast to placeholder relations that feature quantities still in need of further specification: in the context of Newtonian mechanics, $\dot {x} = v$ is a concrete relation, whereas $F = m \ddot {x}$ is a placeholder relation (as the concrete form of *F* still needs to be fixed further in terms of the basic variables *x*,*v* and certain constants; this can for instance be done through choosing $F := \frac {G m_{1} m_{2}}{x^{2}}$ in certain scenarios—cf. Curiel 2016, p. 5).

8 It is in fact through this characterization of the kinematical/dynamical distinction in terms of concrete relations and placeholder relations that Curiel can take the credit for providing its only general clear-cut formulation — despite its omnipresence in the practice of physical theorising.

9 Note, however, that in principle, the ontological interpretation of kinematical structure may itself take various forms, besides the specifically essentialist interpretation considered here. We will come back to this issue in §4.

10 This characterisation goes back to Aristotle, see his *Metaphysics Z.4* for the ‘what it is to be’-formulation and *Topics 102a3* for the link to definition, which has been stressed in the context of contemporary essentialism by Fine (1994a) and others.

11 One might worry here that the kinematical/dynamical distinction is theory-relative, whereas essence and metaphysical modality are not. We will come back to this point later at the end of this section.

12 We will have a bit more to say about the essentialist interpretation at the end of this section, where we in particular discuss a worry tied to the theory-boundness of the kinematical/dynamical distinction.

13 See Folland 2016.

14 It should be clear that our reliance on Kripke here does not mean that we subscribe to the view that all laws of a final physical theory would be metaphysically necessary, a view which is often attributed to him in the literature.

15 Kinematical constraints that would generally clearly count as laws are, for instance, the Pauli exclusion principle (PEP) (as already argued above), and two out the four Maxwell equations. Following Curiel (2016, p. 5) the Maxwell equations **∇**⋅**B** = 0 and $\dot {\mathbf {B}} = - \mathbf {\nabla } \times \mathbf {E}$ are concrete relations in terms of the basic variables **E**, $\dot {\mathbf {E}}$, **B**, and $\dot {\mathbf {B}}$ that hold across all of electrodynamics and are thus kinematical — whereas **∇**⋅**E** = *ρ* and $\dot {\mathbf {E}} = \mathbf {j} - \mathbf {\nabla } \times \mathbf {B}$ contain the placeholder expressions *ρ* and **j** respectively and thus count as dynamical on Curiel’s criterion.

16 We thank an anonymous referee for pressing us to make this clear.

17 To come back to a point raised in footnote 1, note that this gives us a natural way to precisify the otherwise unclear notion of a kind ‘featuring’ in a law (which Tahko uses when stating his account): a kind *k* features in a law of nature *l* iff *l* is true in virtue of the nature of *k*, where ‘is true in virtue of the nature of’ is Fine’s operator which is used in the Essentialist’s canonical formulations of claims about essentiality (see Fine 1994a, b). We merely put this forward as a proposal on Tahko’s behalf, however, and wish to remain neutral about whether this is what Tahko actually has in mind.

## References

[CR1] Armstrong, D. M. (1983). *What is a Law of Nature?*. Cambridge University Press.

[CR2] Bird, A. (2007). *Nature’s Metaphysics*. Oxford University Press.

[CR3] Bird, A. (2012). Are any kinds ontologically fundamental?. In Tahko, T. (Ed.) *Contemporary Aristotelian Metaphysics* (pp. 94–104). Cambridge: Cambridge University Press.

[CR4] Curiel, E. (2016). Kinematics, dynamics, and the structure of physical theory. arXiv:1603.02999.

[CR5] Dretske, F. (1977). Laws of nature. *Philosophy of Science*, 44(2):248–268.

[CR6] Ellis, B. (2001). *Scientific Essentialism*. Cambridge University Press.

[CR7] Fine, K. (1994a). Essence and modality. *Philosophical Perspectives*, 8:1–16.

[CR8] Fine, K. (1994b). Senses of essence. In Sinnott-Armstrong, W., Raffman, D., & Asher, N. (Eds), *Modality, morality and belief. Essays in honor of Ruth Barcan Marcus*. Cambridge University Press.

[CR9] Folland, G. B. (2016). *A course in abstract harmonic analysis*. Chapman and Hall/CRC.

[CR10] Forbes, G. (1989). *Languages of Possibility: An Essay in Philosophical Logic*. Blackwell.

[CR11] Kistler, M. (2002). The causal criterion of reality and the necessity of laws of nature. *Metaphysica*, 3(1):57–86.

[CR12] Kistler, M. (2005). Necessary laws. In *Nature’s Principles* (pp. 201–227). Springer.

[CR13] Kistler, M. (2020). Powers, dispositions and laws of nature. In Meincke, A. (Ed.) *Dispositionalism* (pp. 171–188): Springer.

[CR14] Kripke, S. A. (1980). *Naming and Necessity*. Harvard University Press.

[CR15] Lange, M. (2009). *Laws and lawmakers: Science, metaphysics, and the laws of nature*. Oxford University Press.

[CR16] Lewis, D. K. (1973). *Counterfactuals*. Blackwell.

[CR17] Lewis, D. K. (1986). *On the Plurality of Worlds*. Wiley-Blackwell.

[CR18] Lowe, E. J. (2005). *The Four-Category Ontology: A Metaphysical Foundation for Natural Science*. Clarendon Press.

[CR19] Mellor, D. H., Mellor, D., & et al. (1991). *Matters of metaphysics*. Cambridge University Press.

[CR20] Shoemaker, S. (1980). Causality and properties. In van Inwagen, P. (Ed.) *Time and Cause*. D. Reidel (pp. 109–35).

[CR21] Shoemaker, S. (1998). Causal and metaphysical necessity. *Pacific Philosophical Quarterly*, 79(1):59–77.

[CR22] Sidelle, A. (1989). *Necessity, Essence, and Individuation: A Defense of Conventionalism*. Cornell University Press.

[CR23] Sider, T. (2011). *Writing the Book of the World*. Oxford University Press.

[CR24] Tahko, T. E. (2015). The modal status of laws: In defence of a hybrid view. *Philosophical Quarterly*, 65(260):509–528.

[CR25] Tooley, M. (1977). The nature of laws. *Canadian Journal of Philosophy*, 7(4):667–98.

[CR26] Williamson, T. (2007). *The Philosophy of Philosophy*. Wiley-Blackwell.

